# Identification of key extracellular proteins as the potential biomarkers in thyroid eye disease

**DOI:** 10.1371/journal.pone.0322415

**Published:** 2025-04-29

**Authors:** Shasha He, Han Nie, Xiangbao Yin, Zhiwei Zhong

**Affiliations:** 1 Department of Endocrine and Metabolism, The First Affiliated Hospital of Nanchang University, Nanchang, China; 2 Department of Vascular Surgery, The Second Affiliated Hospital of Nanchang University, Nanchang, China; 3 Department of HepatofIancreatobiliary Surgery, The Second Affiliated Hospital of Nanchang University, Nanchang, China; Rutgers: Rutgers The State University of New Jersey, UNITED STATES OF AMERICA

## Abstract

**Background:**

Thyroid eye disease (TED) is one of the most common autoimmune orbital diseases in adults. The early diagnosis and effective treatment of TED is a worldwide problem. Extracellular proteins may act as indicators in bodily fluids. Our research sought to identify the roles of extracellular proteins and possible biomarkers in TED using a bioinformatics study.

**Methods:**

Data from Gene Expression Omnibus (GEO) were acquired to create the TED expression profiles. The annotation database screened extracellular proteins with differentially expressed genes (EP-DEGs). To investigate both the function and the route of EP-DEGs, GO and KEGG were utilized. Hub genes and protein-protein interaction (PPI) networks among EP-DEGs were discovered. Key EP-DEGs’ diagnostic potency was assessed using the receiver operating characteristic (ROC) curve.

**Results:**

102 EP-DEGs underwent screening. The extracellular matrix, which contains collagen, the receptor-ligand activity, the interaction between cytokines and their receptors, and the complement and coagulation cascades route, were all enhanced in EP-DEGs. The EP-DEG PPI network contained 233 edges and 78 nodes. We discovered 21 extracellular proteins that interacted with EGFR in addition to 3 major extracellular proteins, EGFR, CD44, and CXCL8, all of which had significant values of AUC (> 0.7).

**Conclusions:**

In conclusion, EGFR, CD44, and CXCL8 may be the potential biomarker in the TED. this research gives us a theoretical foundation for understanding how TED pathogenesis occurs.

## 1 Introduction

Thyroid eye disease (TED), also known as thyroid-associated ophthalmopathy (TAO), is one of the most common orbital diseases in adults and belongs to autoimmune diseases [1]. TED is common in hyperthyroidism, but it can also occur in normal or hypothyroidism patients [2]. The primary signs and symptoms include exophthalmos, eyelid contracture, diplopia, corneal ulcers, and vision loss due to optic nerve compression [3]. The disease affects the patient’s appearance, damages eyesight, and brings great inconvenience and pain to patients’ life and work. The disease mainly affects women, with older people and men more likely to develop a severe state [4]. The main pathological changes in TED include cytokine production and inflammation, adipogenesis, hyaluronan synthesis, and myofibrillogenesis [5]. However, the exact pathogenesis of TED is still unclear, which may be closely related to humoral immunity, cellular immunity, abnormal expression of thyrotropin receptor and insulin-like growth factor-1 receptor (IGF-1R) in retrobulbar tissue, race, heredity, and lifestyle [4]. Activated helper T cells bind thyrotropin receptors through thyrotropin receptor antibodies (TRAb), promote the secretion of inflammatory factors and chemokines by ocular fibroblasts, increase the formation of hyaluronic acid and adipogenesis, and eventually lead to ocular tissue remodelings, such as extraocular muscle thickening and ocular adipose hyperplasia [1]. According to the severity of TED, the traditional options are corticosteroids, orbital radiation, and surgical treatment, as well as second-line biologic therapy agents such as rituximab, tocilizumab, and teprotumumab [4]. However, traditional treatments aim to improve symptoms and protect vision, there is no specific treatment for the cause, and second-line drugs are mainly used in North America due to a lack of approval in other countries. In addition, adverse effects have been reported, including hearing loss and cognitive decline as well as recurrence of TED symptoms [4]. Understanding the pathological mechanism of TED will help to discover TED-targeted molecules and provide strong evidence for the future treatment of TED. Therefore, it is necessary to carefully study the molecular mechanism of TED, especially the key genes and pathways related to TED.

Numerous recent investigations have discovered that certain secretory proteins have immunomodulatory effects in the extracellular environment [6–8]. Extracellular proteins may serve as potential indicators or therapeutic targets for TED because they can be found in clinical tissues and various bodily fluids. The popularity of public databases and the rapid advancement of high-throughput sequencing technology have made it possible to classify patients, identify disease subtypes, and discover novel prognostic biomarkers and therapeutic targets that may enable precision medicine. In the study, weighted gene co-expression network analysis was used to create a robust co-expression network with 11 key genes above TED [3]. OSM, CSF3R, CXCL6, DPP4, and PRKCG were useful TED indicators in another investigation based on bioinformatics analysis and clinical validation [9]. Additionally, a study using bioinformatics, proteomics, and miRNA analysis discovered some significant circulating biomarkers, such as catenin, -2 macroglobulin, -2 glycoprotein 1, and fibronectin, which might predict the presence of the TED disease [10]. All of these discoveries point to biomarkers that could aid in examining the biological basis of TED. The study of orbital Extracellular proteins differentially expressed genes (EP-DEGs), which best captures the pathogenic nature of the disease, is, however, lacking in TED.

The Gene Expression Omnibus (GEO) database was utilized to download GSE105149 and GSE58331 concerning TED for this work. We next used the R program to check for DEGs between TED orbital tissue normal samples. We separated the EP-DEGs from the DEGs using screening. Then, the Kyoto Encyclopedia of Genes and Genomes (KEGG) pathways and Gene Ontology (GO) terms were used to analyze the biological enrichment of these proteins. To identify common hub genes and extracellular molecules interacting with hub genes, protein-protein interaction (PPI) network analysis was carried out. Finally, receiver operating characteristic (ROC) curves are used to evaluate the diagnostic ability of the key EP-DEG. In this study, the main extracellular protein that may contribute to the pathogenesis of TED will be identified, and new treatment targets will be investigated.

## 2 Materials and methods

### 2.1 Acquisition and processing of data

The most comprehensive global database for gene expression and microarray chips is GEO (http://www.ncbi.nlm.nih.gov/geo). Based on the GPL570 platform, the GEO database was used to download two gene expression profiles (GSE105149 and GSE58331). GSE105149 and GSE58331 were selected based on their relevance to TED pathology. GSE58331 includes orbital tissue samples (n=64), which directly reflect TED-associated changes in the affected tissue. GSE105149 contains lacrimal gland samples (n=11), chosen to explore potential systemic biomarkers. Both datasets utilize the GPL570 platform, ensuring consistency in microarray processing. The merged analysis aimed to increase statistical power and capture both localized and systemic extracellular protein profiles. Batch effects between GSE105149 and GSE58331 were minimized using ComBat normalization. Tissue source (orbital vs. lacrimal gland) and disease severity were noted as potential confounders but were addressed by stratified analysis. The gene expression levels of 64 anterior orbit samples from TED patients (n = 35) and healthy individuals (n = 29) are included in the GSE58331 dataset, along with the gene expression levels of 11 lacrimal gland samples from TED patients (n = 4) and healthy individuals (n = 7), in the GSE105149 dataset. Based on Illumina’s annotation documentation, probes were mapped to the recognized gene symbols.

### 2.2 DEGs and EP-DEGs screening

A potent technique for examining DEGs is the limma package in R [11]. In the GSE105149 and GSE58331 datasets, we filtered DEGs between TED and control The threshold values for DEGs detection were adj. P < 0.05 and |log_2_fold change (FC)| ≥ 0.5. Then, using the Human Protein Atlas (HPA) and the UniProt database, we obtained the extracellular protein gene list GO:0005615 [12,13]. The DEGs connected the two lists. The common gene discovered using these two approaches was designated as EP-DEGs, and we examined its differential expression in the TED group compared to the control group.

### 2.3 Analysis of EP-DEGs’ Functional Enrichment and Pathways

To better understand the biological significance of EP-DEGs, we used the Database for Annotation, Visualization, and Integration Discovery (DAVID; https://david.ncifc rf.gov/) to perform GO and KEGG pathway enrichment analyses for the EP-DEGs. The categories for GO analysis are biological process (BP), cellular component (CC), and molecular function (MF). Statistical significance was set at P < 0.05. We performed a KEGG pathway enrichment analysis after classifying EP-DEGs into up-regulated and down-regulated groups.

### 2.4 Gene expression analysis and the construction of the EP-DEGs PPI Network

The relationship of EP-DEGs was examined using the STRING online database’s protein-protein interaction (PPI) network and visualized using Cytoscape software (version 3.9.0). To identify functional clusters of genes in the PPI network, the Cytoscape tool Molecular Complex Detection (MCODE) was used. The top 10 modules with more hub genes were screened out. We used the CytoHubba plugin to find the top 10 key genes in 10 ways, then we chose the top 5 hub genes with the most occurrence in different algorithms for constructing ROC curves.

### 2.5 Statistical analysis

The ROC curve was constructed by SPSS22.0, and the area under the curve (AUC) of the hub gene was calculated. The area under the ROC curve is between 0.5 and 1. The closer the AUC is to 1, the better the diagnostic effect is. AUC has a lower accuracy in the interval of 0.5–0.7, a certain accuracy in the interval of 0.7–0.9, and a high accuracy above 0.9.

## 3 Results

### 3.1 Identification of DEGs

**Fig 1:**
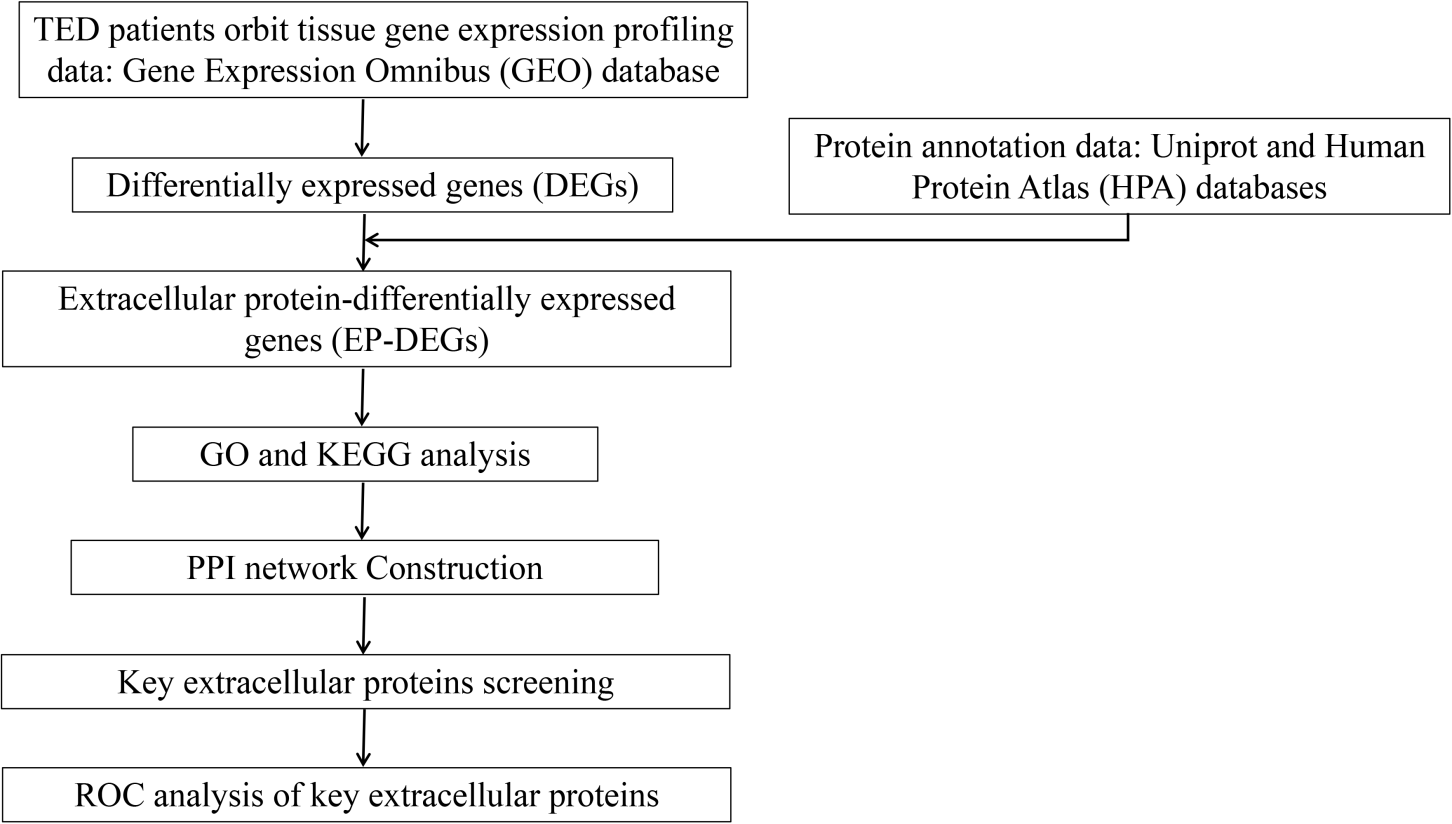
Flow chart of the study.

**Fig 2:**
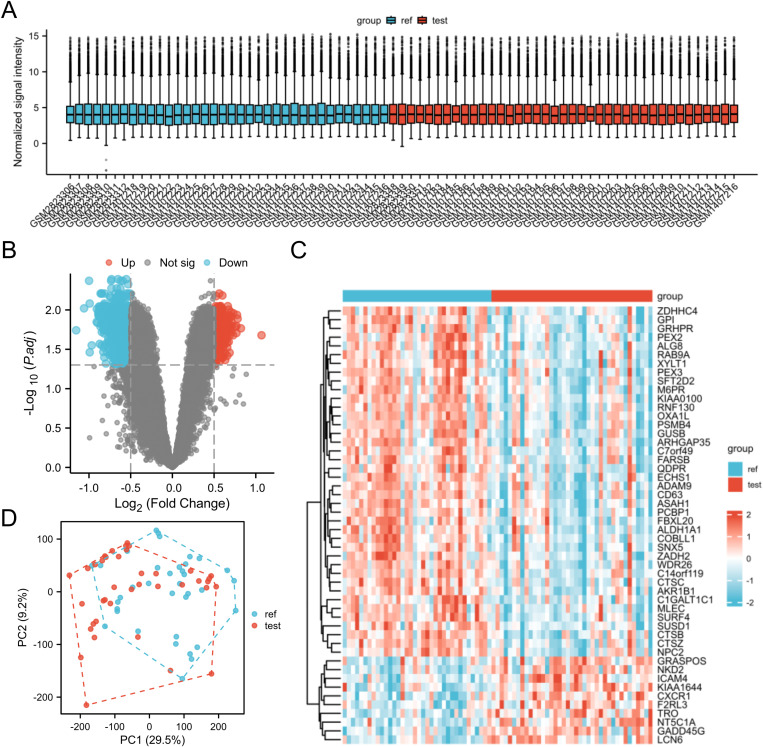
Analyze the dataset’s differences in gene expression (DEGs) between the TED group and the control group. (A) Gene probe expression levels in a box plot across samples. (B) TED group and control group DEGs on a volcano map (adj. P < 0.05 and |log2fold change (FC)| ≥ 0.5). (C) Heatmap of high and low expression of top 20 genes in TED group and control group. (D) Principal-component analysis (PCA).

The study design is shown in Fig 1. The median of each sample is basically on a horizontal line, with a good degree of normalization between samples (Fig 2A). There were 1160 down-regulated genes and 465 up-regulated genes in the volcano map (Fig 2B). A heatmap of the top 25 genes that were up- and down-regulated was displayed (Fig 2C). The first principal component (PC1) accounted for 29.5%, according to principal component analysis (Fig 2D).

### 3.2 Screening of EP-DEGs

**Fig 3:**
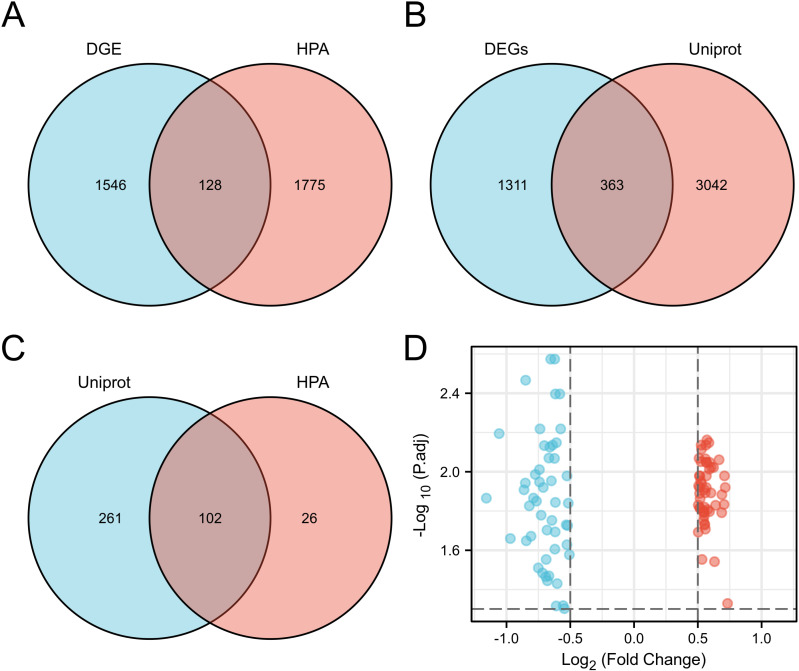
Screening extracellular proteins differentially expressed genes (EP-DEGs). (A) EP-DEGs annotated in HPA. (B) EP-DEGs annotated in UniProt database. (C) The overlapping EP-DEGs were screened by two databases. (D) Volcanic map of EP-DEGS.

DEGs identified in Fig 2 were cross-referenced with extracellular protein databases (UniProt/HPA) to derive EP-DEGs for downstream analyses. Regarding the annotated extracellular protein gene in the public library, 128 EP-DEGs were screened from the HPA database (Fig 3A), and 363 EP-DEGs were screened from the UniProt database (Fig 3B). 52 up-regulated and 50 down-regulated EP-DEGs were selected from 102 EP-DEGs acquired from these two databases (Figs 3C and 3D).CHAD, SCUBE1, LTBP3, ANGPTL7, IL6, S100A12, NAPSA, CFB, GPHA2, and CXCL8 were the top 10 up-regulated EP-DEGs with the least adj. P-values and CP, CFH, TF, EFEMP1, DPP4, ADAM9, F13A1, CD55, SFRP1, and PLA2G2A were the top 10 down-regulated EP-DEGs (Table 1).

**Table 1:** The top 10 up-regulated and down-regulated EP-DEGs in TED human orbit tissue (GSE58331 and GSE105104).

Gene.Symbol	logFC	AveExpr	P.Value	adj.P.Val
CHAD	0.73323569	4.89505127	0.014238021	0.046886307
SCUBE1	0.714575715	7.035388669	0.000780607	0.012023422
LTBP3	0.708773426	6.407440097	0.000509644	0.010495533
ANGPTL7	0.705785566	4.387759944	0.001531073	0.014677022
IL6	0.688803569	4.392241996	0.001107114	0.013083282
S100A12	0.687230659	2.834742589	0.001932542	0.016164847
NAPSA	0.666538499	4.905533201	0.000235932	0.008688954
CFB	0.63943685	5.604495481	0.001569674	0.014811023
GPHA2	0.630830182	3.606790652	0.00624768	0.028706454
CP	-1.158050788	6.630619217	0.001240586	0.01362961
CFH	-1.057709186	6.421517581	4.15E-05	0.006392091
TF	-0.969683052	6.774118598	0.003752407	0.021888561
EFEMP1	-0.862729612	8.833440808	0.000869459	0.0123595
DPP4	-0.852392592	6.718226482	0.000657382	0.011415891
ADAM9	-0.850046533	6.113296	3.19E-06	0.00341638
F13A1	-0.844882865	6.821091446	0.003947686	0.022474812
CD55	-0.821223992	8.213376396	0.0015962	0.014916256
SFRP1	-0.807776985	8.577555313	0.003571532	0.021294473
PLA2G2A	-0.788547828	9.966741748	0.001234042	0.013620374

### 3.3 Analysis of EP-DEGs’ enriched pathways using GO and KEGG

**Fig 4:**
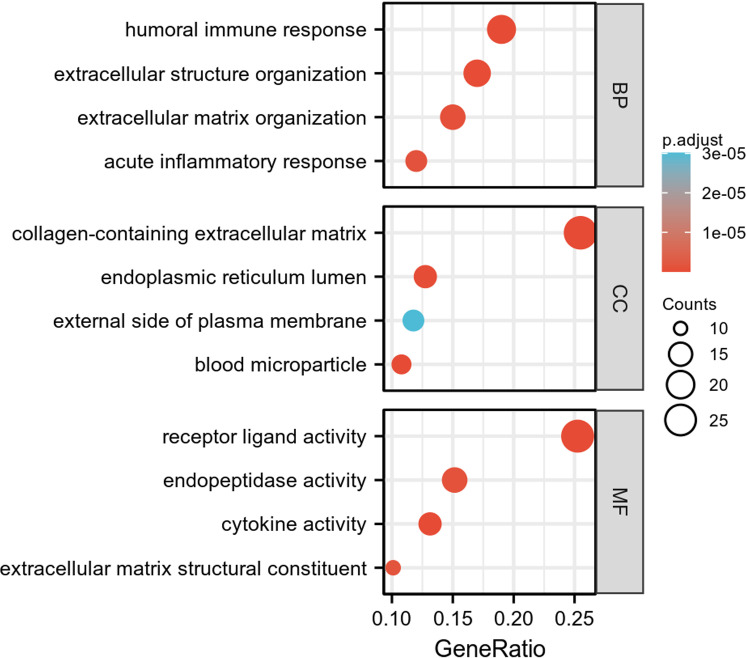
GO enrichment analysis of EP-DEGs in BP, CC, and MF.

**Fig 5:**
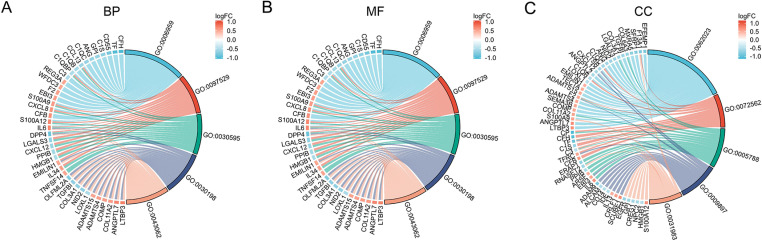
Chord diagram of EP-DEGs in BP, CC, and MF (A-C).

**Fig 6:**
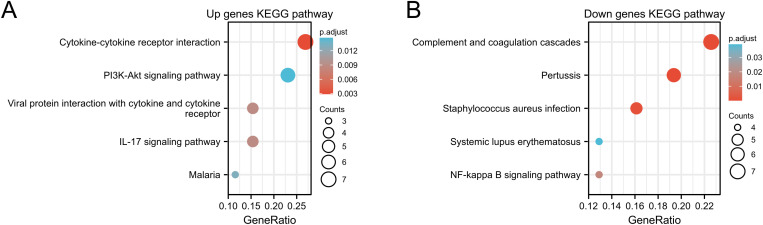
KEGG enrichment analysis of up-regulated and down-regulated EP-DEGs (A, B).

GO enrichment and KEGG pathway analysis compared TED and control samples to investigate the role of EP-DEGs in TED. EP-DEGs were mainly enriched in humoral immune response, extracellular structure organization of BP, the collagen-containing extracellular matrix of CC, and the activity of MF’s receptor ligands (Fig 4). The enriched GO items for BP, MF, and CC are shown in the chord diagram correspondingly (Figs 5A-C). In KEGG pathway analysis, the up-regulated genes are enriched in cytokine-cytokine receptor interaction, IL-17 signaling pathway, viral protein interaction with cytokine and cytokine receptor, and PI3K-Act signaling pathway, and down-regulated genes are concentrated in the staphylococcus aureus infection, pertussis, complement and coagulation cascades, and NF-kappa B signaling pathway (Figs 6A and B).

### 3.4 Creation of the PPI network and determination of hub genes

**Fig 7:**
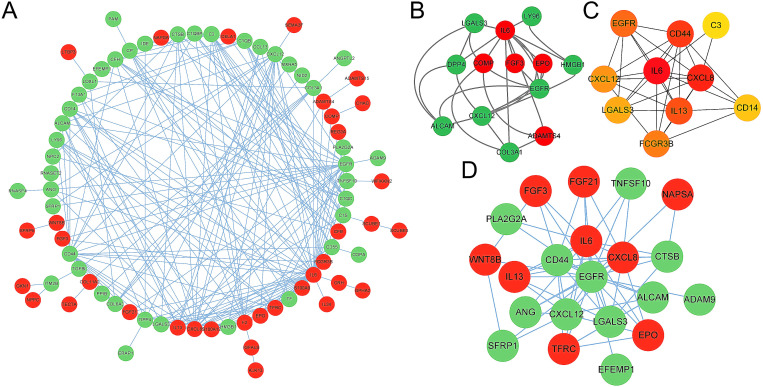
Network construction for EP-DEGs and hub gene screening. (A) Creating the EP-DEG PPI network using STRING data. (B) The MCODE plug-in for Cytoscape created the node gene cluster. (C) CytoHubba was used to construct the central gene of Top10 by the MCC algorithm. (D) The first node gene that interacted with EGFR was predicted by CytoHubba.

The STRING database was utilized to build a PPI network comprising 102 EP-DEGs to comprehend the interactions between the proteins that correspond to EP-DEGs. The PPI network was viewed using the Cytoscape program (Fig 7A). The red node represented the up-regulated EP-DEGs, and the green node represented the down-regulated EP-DEGs. The PPI network has 233 edges and 78 nodes (nodes=genes, edges=interactions). The red color represented up-regulated EP-DEGs, and the green color represented down-regulated EP-DEGs (red=up-regulated, green=down-regulated). The MCODE plug-in in Cytoscape is used for functional modules. Two clusters were identified by MCODE analysis. Cluster 2 contains more top 10 genes than cluster 1, we extracted cluster 2, which has 13 genes and 28 edges (Fig 7B). The top 10 genes are screened using the CytoHubba application’s 10 algorithms. The top five genes with the most occurrence within different algorithms were IL6, CD44, CXCL8, EGFR, and CXCL8, and EGFR was shown in all 10 methods (Table 2). The top 10 hub genes were identified by the MCC methods using the CytoHubba plug-in of Cytoscape (Fig 7C). Create the first gene that communicates with EGFR using CytoHubba. Nine genes are up-regulated, and twelve are down-regulated out of 21 genes examined (Fig 7D).

**Table 2:** The top10 EP-DEGs by 10 topological analysis methods of cytoHubba.

MCC	Degree	EPC	BottleNeck	EcCentricity	Closeness	Radiality	Betweeness	Stress	MNC
IL6	IL6	IL6	IL6	ALCAM	IL6	IL6	IL6	IL6	IL6
CXCL8	CXCL8	CXCL8	CD44	LGALS3	CXCL8	CD44	COL3A1	CD44	CXCL8
CD44	CD44	CD44	COL3A1	CD14	CD44	CXCL8	CD44	EGFR	CD44
IL13	EGFR	EGFR	CXCL12	DPP4	EGFR	CXCL12	EGFR	COL3A1	EGFR
EGFR	CXCL12	CXCL12	EGFR	CTSB	CXCL12	EGFR	CXCL12	CXCL8	CXCL12
FCGR3B	CD14	FCGR3B	CFB	TFRC	COL3A1	LGALS3	TGFBI	CXCL12	CD14
CXCL12	TFRC	IL13	COL6A3	CD44	TFRC	TFRC	CXCL8	C1S	TFRC
LGALS3	C3	TFRC	CTSB	EGFR	LGALS3	COL3A1	F2	F2	C3
CD14	FCGR3B	CD14	TGFBI	IL13	F2	CTSB	C1S	CFH	FCGR3B
C3	COL3A1	LGALS3	F2	FCGR3B	FCGR3B	IL13	ITM2B	TGFBI	IL13

### 3.5 Validation of diagnostic value of hub genes

**Fig 8:**
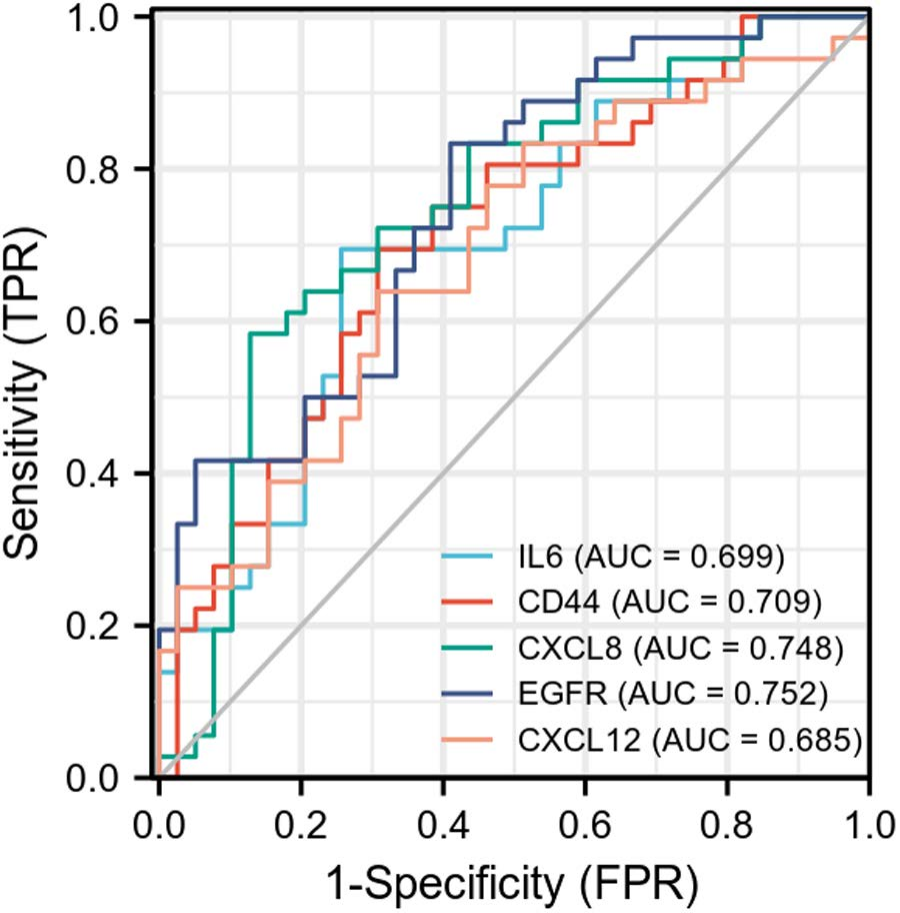
Receiver operating characteristic (ROC) curves were used to evaluate the top 5 EP-DEGs with the most occurrence in different algorithms.

To validate the diagnostic value of the top 5 hub genes with the most occurrence in different algorithms, we constructed ROC curves and calculated the AUC corresponding to the expression levels of these genes in the data set. Only 3 genes have certain accuracy (AUC > 0.7). The AUC for IL-6, CD44, CXCL8, EGFR and CXCL12 were 0.699 [95% confidence interval (CI), 0.579–0.818], 0.709 (95% CI, 0.591–0.827), 0.748 (95% CI, 0.634–0.862), 0.752 (95% CI, 0.643–0.861), and 0.685 (95% CI, 0.564–0.807) (Fig 8).

## 4 Discussion

### 4.1 EP-DEGs may be involved in the biological process of TED

We analyzed the GSE105149 and GSE58331 datasets and got 1674 DEGs. In the UniProt and HPA databases, we compared DEGs with a list of extracellular protein genes. As opposed to 26 genes that did not overlap, 102 EP-DEGs in the HPA database did. According to the GO enrichment assay, EP-DEGs were highly abundant during a humoral immune response, extracellular structure, collagen-containing extracellular matrix, and receptor-ligand activity. Extracellular proteins are thought to play a role in cellular signal transduction, drug transport, humoral immune responses, and cellular immune responses in the pathogenic process of TED. The Chord maps revealed that EP-DEGs was involved in a variety of biological processes and were rich in GO keywords. The PI3K-Act signal pathway and cytokine-cytokine receptor interaction were where the up-regulated EP-DEGs were most concentrated, according to KEGG enrichment analysis. The study showed that the thyroid stimulating hormone receptor (TSHR) signal promoted the proliferation of orbital fibroblasts through PI3K/Akt signal pathway in TED [14]. It implies that TED extracellular protein may primarily be a few cytokines that activate pathways in the immune system. The complement and coagulation cascade were where the down-regulated EP-DEGs were most concentrated. The complement and coagulation cascade signaling pathway are one of the most abundant functional pathways in bioinformatics research. The disorder of this pathway is usually caused by innate dysfunction of the immune system [15]. These findings imply that signaling pathways for complement and the coagulation cascade may control the inflammatory response in TED.

### 4.2 The main extracellular protein involved in the pathophysiology of TED may be EGFR

The top five hub genes with the highest frequency in various techniques after studying the PPI network of EP-DEGs were EGFR, CD44, CXCL8, CXCL12, and IL-6. Among them, CXCL8 and IL-6 were up-regulated genes, and CD44, CXCL12, and EGFR were down-regulated genes. We discovered that EGFR was concurrently present in the clutter of MCODE and the top 10 hub genes assessed by 10 CytoHubba topological approaches. These three genes (EGFR, CD44, and CXCL8) had good accuracy as biomarkers for diagnosing TED. EGFR is widely distributed on the surface of many cell membranes, such as epithelial cells, fibroblasts, glial cells, tumor cells, and so on [16]. CD44 is a kind of cell adhesion molecule that mediates cell-cell adhesion or cell-extracellular matrix adhesion [17]. There were few studies on EGFR and CD44 in TED. Higher levels of EGFR and CD44 were observed in the TED group [18], yet our analysis revealed that the genes’ expression was down-regulated. The difference may be due to the severity of the disease and the size of the sample. There were only 3 cases of moderate to severe TED [18], compared with 39 cases of TED enrolled in our study, but the severity of the disease was not reported. Studies have found that LncRNA LPAL2/miR-1287-5p/EGFR/PI3K/AKT axis regulates the activation of TED-derived orbital fibroblasts through cell adhesion factor [18,19]. CXCL8, also known as interleukin-8 (IL-8), is a cytokine of the chemokine family, mainly produced by epithelial cells and macrophages. Studies have shown that IL-8 is highly expressed in TED, acts on immune cells, aggravates inflammatory response, and makes orbital fibroblasts secrete extracellular matrix, proliferate and differentiate into myofibroblasts or lipid fibroblasts, leading to tissue fibrosis and orbital remodeling [20]. CXCL12, a chemokine distinct from IL-12, was downregulated in TED and may regulate leukocyte infiltration [21]. Our study found that IL-6 was distinctly increased in TED, suggesting that the inflammatory proteins may play an important role in the pathogenesis [22]. Tocilizumab, an IL-6 receptor monoclonal antibody, is considered a good alternative in corticosteroid-resistant TED. The mechanism may change the gene expression of fibroblasts through different pathways [22]. Although the role of IL-6 has been researched by many studies [20–24], the accuracy of IL-6 as a biomarker of TED in our study was low, and more studies are needed to prove it. p38 MAPK activation promoted IL-6 production [25].Of all the genes, EGFR has the highest diagnostic accuracy. Therefore, As CD44 is a crucial adhesion molecule and CXCL8 is a crucial cytokine, we hypothesize that EGFR may be the major extracellular protein in the pathogenesis of TED. However, EGFR-targeted therapy mainly focuses on tumors [26–28]. For a clinical application for TED, EGFR still needs to be further investigated. While prior studies reported upregulated EGFR in TED fibroblasts, our analysis showed downregulation. This discrepancy may arise from differences in tissue types (orbital vs. fibroblast cultures) or disease stages (acute vs. chronic). Future studies should stratify patients by disease severity to clarify EGFR’s role.

### 4.3 Mechanistic and clinical implications

#### 4.3.1 Biological pathways.

The PI3K/Akt pathway, linked to TSHR signaling, may drive fibrosis via extracellular matrix remodeling. Complement cascade downregulation suggests dysregulated immune tolerance, contributing to chronic inflammation.

#### 4.3.2 Clinical translation.

EGFR, CD44, and CXCL8 show diagnostic potential but require validation in serum/tear samples. Challenges include biomarker specificity and standardization across platforms.

## 5 Limitations

This research has several restrictions. Data on gene expression in human orbital tissue are currently scarce, which could lead to inconsistent findings. Two protein annotation databases are used to screen extracellular proteins, although most of the information about protein localization in both annotation databases originates from published research. The evidence is lacking, and it’s possible that certain extracellular proteins were overlooked. The small sample size in GSE105149 (n=4 TED vs. n=7 controls) may reduce statistical power. Future studies with larger cohorts are needed to validate these findings. While bioinformatics findings are predictive, experimental validation (e.g., immunohistochemistry for EGFR/CD44/CXCL8 in TED orbital tissues) is essential. Collaborative efforts to access clinical samples are underway.

## 6 Conclusions

In this study, 102 EP-deg were screened, and the gene expression profile of TED human orbital tissue was evaluated. We foresee the biological routes and processes that they take part in. In addition, 3 keys and 21 extracellular proteins that may interact with EGFR are predicted by us. The aim of this work is to give bioinformatics for the identification of potential TED biomarkers.

## Supporting information

S1 Table EP-DEG screening data. (XLSX)
